# Interpretation of Near-Infrared Imaging in Acute and Chronic Wound Care

**DOI:** 10.3390/diagnostics11050778

**Published:** 2021-04-26

**Authors:** Jonathan Arnold, Valerie L. Marmolejo

**Affiliations:** 1 Medical Director, Mercy Healing Center, 701 10th Street SE, Ground Floor, Cedar Rapids, IA 524031, USA; 2 Scriptum Medica, Medical Writing, University Place, WA 98466, USA; VLSDPM@GMAIL.COM

**Keywords:** inflammation, local ischemia, collateralization, hyperbaric oxygen therapy

## Abstract

Vascular assessment is a critical component of wound care. Current routine noninvasive vascular studies have limitations which can give a false sense of security of the presence of adequate perfusion for healing. Near-infrared imaging modalities can serve as an additional diagnostic assessment of wounds in which adequate perfusion is a concern. Correct interpretation of near-infrared images obtained is critical as subtleties that exist in the acute and chronic wound population goes beyond the interpretation that increased signal is consistent with adequate perfusion for healing. The objective of this paper is to educate providers on the correct interpretation of this point-of-care imaging modality in day-to-day wound-care practice to guide clinical decision-making for rapid wound resolution.

## 1. Introduction

Acute and chronic wound care involves clinical decision-making based on a thorough history and physical examination and interpretation of laboratory, imaging, and other diagnostic tests performed to guide treatment toward timely resolution. Vascular assessment to determine if adequate perfusion exists for healing is critical in this process. This is particularly important for wounds that occur on the lower extremity. Approximately 8.5 million people in the US over the age of 40 have peripheral arterial disease [1]. This estimate may be low more as over 50% of asymptomatic patients go undiagnosed [2] Providers must have a high index of suspicion to prompt lower extremity vascular assessment beyond history and physical examination findings to ensure peripheral arterial disease is not a contributing factor to delayed healing [3].

The most common test obtained, if lower extremity vascular assessment via history and physical examination findings are concerning for peripheral arterial disease, involves comparison of ankle and toe pressure measurements to those of the upper extremity, the ankle brachial and toe brachial index, respectively. Results of these tests are limited given that perfusion assessment is localized to the vessel being examined at the level of cuff placement only. Results obtained must be extrapolated to whether this equates to adequate perfusion for healing of a more distal wound. Falsely elevated results can also occur due to factors such as vessel calcification, collateralization, tobacco use, and caffeine intake [1,4,5,6,7,8,9,10,11]. Falsely elevated results are often seen in patients with critical limb ischemia, end-stage renal disease, and diabetes; patients prone to lower extremity ulceration [4,10,11]. Over 50% of these patients have peripheral arterial disease as a contributing factor to delayed healing. When this is undiagnosed and untreated, delayed wound healing persists, leading to a greater risk of infection, amputation and death [1,2,4,5].

When ankle and toe pressure measurements are inconclusive, an additional vascular study, transcutaneous oxygen pressure measurements (TCOM), may be employed. Oxygen is an essential component in fibroblast proliferation, collagen synthesis and epithelization [12,13]. TCOM results have been reported to better predict wound healing potential and the necessity for amputation compared to the ankle brachial index [6]. However, this study has limitations as well, namely the ability to provide oxygen pressure measurements at the site of probe placement only and the adverse effects on results obtained due to edema, dry flaky skin, maceration, callused or plantar skin, cellulitis, and improper probe placement over bones and tendons [14]. The test also requires skilled personnel to properly perform the test. The test is also time and labor intensive [7,9,14,15]. 

Novel near-infrared imaging techniques, which allow visualization of site-specific tissue perfusion and/or oxygenation saturation levels, have recently emerged as a potential modality to supplement routine noninvasive vascular studies in vascular assessment. Near-infrared imaging can be performed in two ways: (1) fluorescence angiography (SPY Elite and SPY-PHI, Stryker, Kalamazoo, MI) and (2) non-contact, noninvasive, handheld near-infrared imaging devices that provide tissue oxygenation saturation levels based on the oxygen-carrying status of hemoglobin (Snapsho2tNIR, Kent Imaging, Calgary, AB Canada). Fluorescence angiography is an invasive imaging method that provides dynamic and static information on local tissue perfusion. It involves injection of a fluorescing dye, the two most common being fluorescein and indocyanine green. The shorter half-line, hepatic clearance, ability of the dye to remain intravascular and low adverse reaction rate has led to the use of indocyanine green over fluorescein. Indocyanine green fluorescence angiography is used in the operating room for intraoperative vascular assessment in general, plastic and colorectal surgery and has been used in the outpatient setting for perfusion assessment of wounds [7,8,16,17,18,19,20]. The size of the device and need for intravenous access hinders its use in the outpatient setting. Non-contact, noninvasive, handheld devices have fostered increased use of near-infrared imaging in the outpatient setting given its portable, rapid assessment and noninvasive nature. Image capture of the wavelengths of light returned and analyzed by the device, based on the oxygen-carrying status of hemoglobin, allow for site-specific assessment of the tissue perfusion and oxygenation saturation levels [7,8,16,20].

Near-infrared imaging results are not affected in the same way by the limitations that can make routine noninvasive vascular study results unreliable or inconclusive. The ability to determine tissue perfusion and oxygenation levels directly at the site of an acute or chronic wound with the objective, point-of-care, site-specific modality near-infrared imaging provides can aid in clinical decision-making for expedited wound resolution. Direct visualization of the area of interests allows the provider to determine the:

- Effects of factors that are known to delay wound healing;
- Impact revascularization plays directly on the wound;
- Adequacy of wound debridement;
- Response to advanced wound-care modalities (i.e., hyperbaric oxygen therapy, negative pressure wound therapy);
- Proper timing of placement of biologics to aid in wound healing; and
- The most distal level of amputation most likely to primarily heal [8,16,17,21,22,23].

Proper interpretation of the images received with near-infrared imaging modalities in acute and chronic wound care is critical in guiding clinical decision-making. The clinician must understand how imaging in this setting varies and the factors that can affect the results obtained. Through years of experience and a multitude of image review of both invasive and noninvasive near-infrared imaging modalities from different facilities and clinicians, the authors have been able to discern characteristic imaging patterns for factors that can contribute to delayed healing and those that are positive prognostic indicators that a wound is responding to treatment and moving toward resolution. Proper interpretation of these images can help guide clinical decision-making for expeditious and optimal patient outcomes. The objective of this paper is to educate providers on correct interpretation of this point-of-care imaging modality in day-to-day wound-care practice and assessment.

## 2. Guide to Near Infra-Red (NIR) Image Interpretation

Initial studies on invasive near-infrared imaging modalities focused on tissue perfusion assessment following revascularization [8,17,21,22,23,24,25]. These devices work through a binary type of image interpretation in which increased fluorescence signal correlates with increased tissue perfusion. This generic image interpretation carried over into the use of fluorescence angiography for imaging of acute and chronic wounds. However, subtleties exist in these patients that cause this basic method of image assessment to not hold true. These subtleties can cause images to be interpreted as presence of adequate tissue perfusion and oxygen levels necessary for healing to occur when that is not the case.

### 2.1. Factors Contributing to Delayed Wound Healing

#### 2.1.1. Chronic Wound Stalling in the Inflammatory Phase of Wound Healing

**Figure 1:**
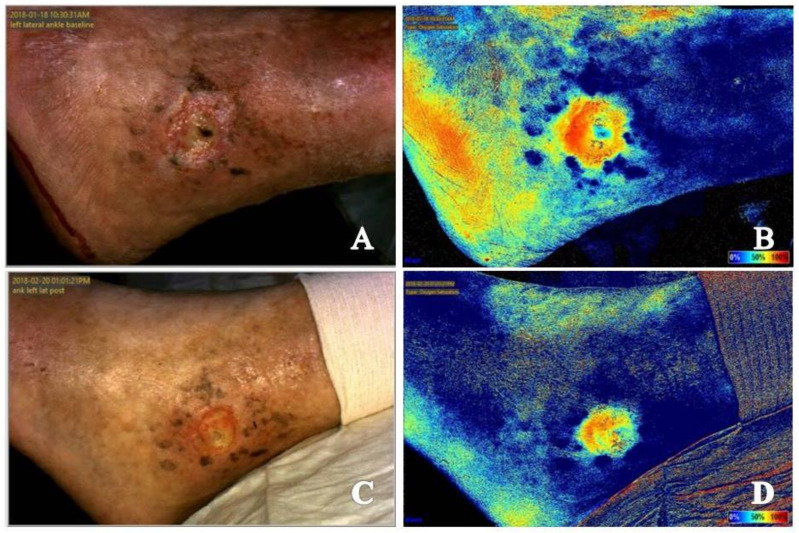
(**A**) Baseline clinical photograph; and (**B**) near-infrared image assessment of the left lateral ankle. Note on the near-infrared image the increased signal (color closer to 100% on rainbow scale on near-infrared image) in the periwound area and reduced signal with the wound bed, indicative of a chronic wound stalled in the inflammatory phase of wound healing. The mottled signal pattern seen about the wound is indicative of the presence of local ischemia. Increased signal noted in the lateral aspect of the foot in the location of the fifth metatarsal signals potential underlying infection. The patient had confirmed diagnosis of osteomyelitis of the fifth metatarsal. (**C**) Clinical photograph; and (**D**) near-infrared image assessment following vascular intervention. Note the reduction of signal within the periwound area and increased signal within the wound bed in the near-infrared image following revascularization. The reversal of signal from the baseline image is a positive prognostic indicator that the wound has transitioned to the proliferative phase of healing. Reduction in signal intensity in the area of the fifth metatarsal is evidence of positive response to treatment of underlying osseous infection.

**Figure 2:**
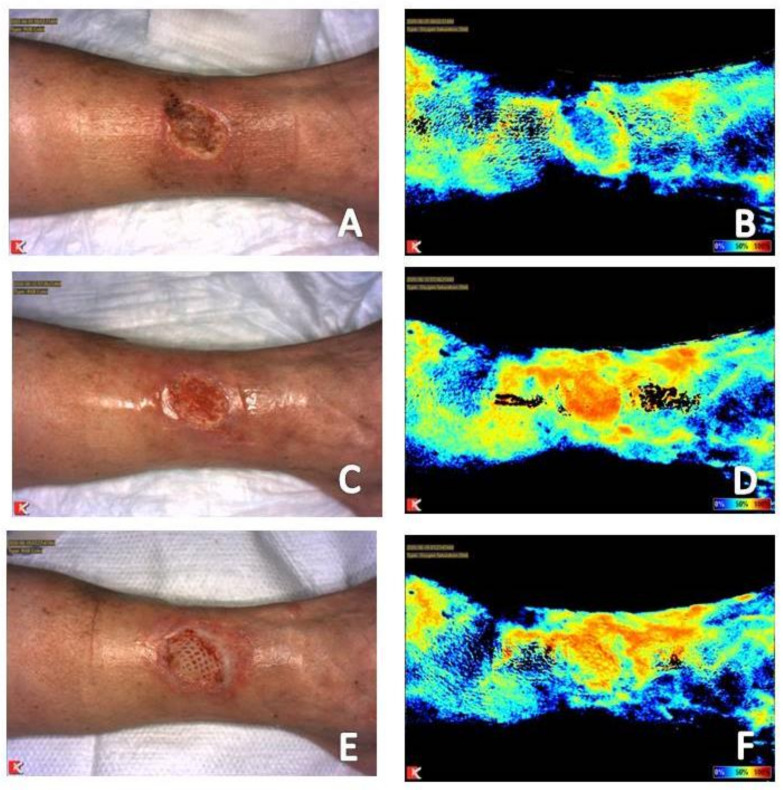
(**A**) Baseline clinical photograph; and (**B**) near-infrared image assessment of the right lateral leg. Note on the near-infrared image the increased signal (color closer to 100% on rainbow scale on near-infrared image) in the periwound area and reduced signal with the wound bed, indicative of a chronic wound stalled in the inflammatory phase of wound healing. (**C**) Clinical photograph; and (**D**) near-infrared image assessment following initiation of compression therapy. Note the reduction of signal within the periwound area and increased signal within the wound bed in the near-infrared image following initiation of compression therapy. A human acellular dermal matrix was applied when angiogenesis was evident within the entire wound bed on near-infrared imaging assessment two weeks after placement, (**E**,**F**).

Multiple studies have shown that a chronic wound stalled in a dysfunctional, inflammatory phase of wound healing presents as a wound with increased signal in the periwound area and reduced signal within the wound bed (Figure 1A,B and Figure 2A,B) [19,20,26]. One can easily understand how the presence of signal consistent with increased perfusion and tissue oxygenation saturation levels around the wound can easily be misinterpreted as adequate vascular supply for healing. However, the dynamics of what is occurring at the microvasculature level around a chronic wound must be considered. In the inflammatory phase of healing, collateral vessels around the wound dilate in attempts to provide adequate nutrients and oxygen for healing to occur. However, the ability for oxygen to enter the wound bed is hindered, resulting in a hypoxic wound bed [7,20]. Thus, explaining the characteristic appearance of chronic wounds on both types of near-infrared imaging techniques.

#### 2.1.2. Local Ischemia

**Figure 3:**
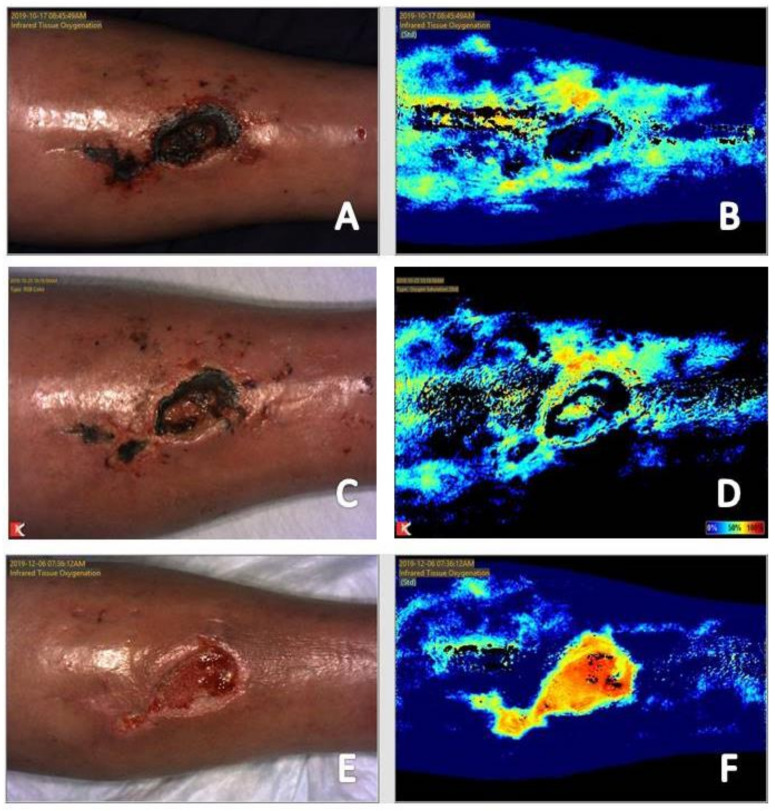
(**A**) Baseline clinical photograph; and (**B**) near-infrared image assessment of the left anterior leg taken prior to debridement and vascular intervention (17 October 2020). Note absence of signal in the wound bed, secondary to stable eschar in place, and dilation of surrounding collateral vessels, proximal to the wound bed, attempting to supply the wound bed. Note the increase in signal in the wound bed upon debridement of loose eschar within the wound bed (**C**,**D**). Clinical photograph (**E**) and near-infrared image (**F**) following vascular intervention. Note increase of signal in the wound bed with reduction in signal of surrounding collateral vessels. This reversal of signal following vascular intervention is a positive prognostic indication of progression of a wound towards resolution.

Local ischemia around a wound appears as reduced signal to the wound bed with a mottled signal appearance in the periwound area (Figure 1 and Figure 3). The mottled appearance is thought to be secondary to dilation of collateral vessels in attempts to supply the wound bed, resulting in shunting from other vessels with corresponding reduction in signal appearance [7,19,27]. This mottled appearance will improve or resolve with appropriate vascular intervention [7,27].

#### 2.1.3. Infection

**Figure 4:**
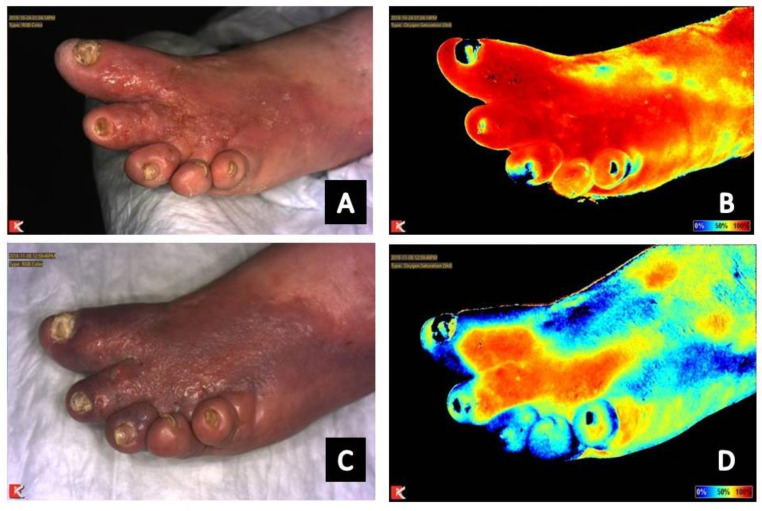
(**A**) Baseline clinical photograph; and (**B**) near-infrared image assessment of the left dorsal foot. Note on the near-infrared image the increased signal in signal on the dorsum of the foot secondary to skin and soft tissue infection. (**C**) Clinical photograph; and (**D**) near-infrared image assessment following initiation of antibiotic therapy. Note the reduction of signal on the dorsal foot following initiation of antibiotic therapy.

**Figure 5:**
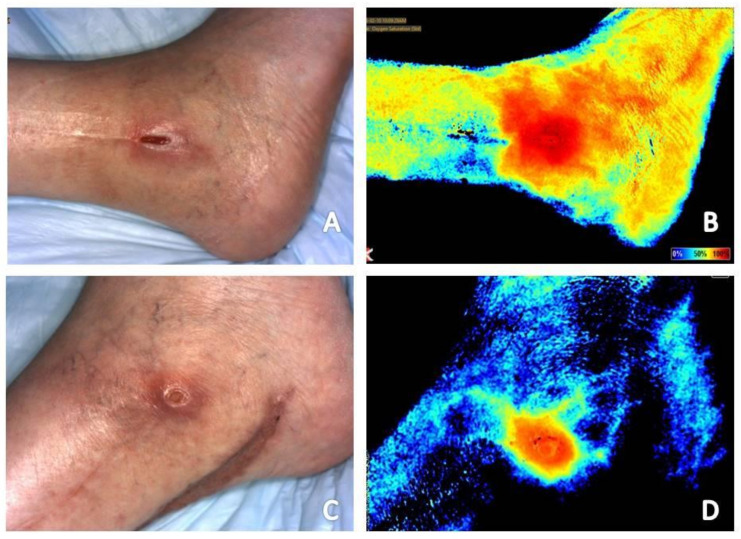
(**A**) Baseline clinical photograph; and (**B**) near-infrared image assessment of the right lateral ankle. The patient had undergone open reduction and internal fixation of an ankle fracture which was subsequently complicated by osteomyelitis. Note on the baseline near-infrared image (**B**) the increased signal in the area of the distal fibula incorporating and extending beyond the wound bed. (**C**) Clinical photograph; and (**D**) near-infrared image assessment following surgical intervention consisting of debridement, skin and soft tissue flap for closure, and antibiotic therapy. Note the reduction of signal on the near-infrared image, with localization only to the area of the wound, indicting resolving infection and a wound in the proliferative phase of healing.

Infection, whether of the skin and soft tissue or underlying bone, presents as intense and retained signal in the area of infection (Figure 1B,D, Figure 4A–D and Figure 5A–D) [7,19]. Positive response to treatment corresponds with reduction in signal to the area over the course of serial near-infrared imaging assessment. Again, the dynamics of what is occurring at the local microvasculature level must be considered. Local vessels dilate to supply the site with the nutrients and cells required to combat the infection resulting in increased signal to the area with subsequent reduction as the infection resolves.

### 2.2. Factors Resulting in Variations in Tissue Thickness—Edema, Atrophic Tissue, Tissues, and Products within the Wound Bed

The depth of tissue penetration with near-infrared imaging ranges from 2 mm to 10 mm [19]. Thickness of the subcutaneous tissue can affect the depth of penetration and the resultant images obtained [28,29,30]. Increased tissue thickness will attenuate the resultant signal reading obtained while decreased tissue thickness will do the opposite [19]. An example of a condition resulting is increased tissue thickness is lower extremity edema, a key factor that plays a role in delayed healing of venous leg ulcerations [31]. Increased tissue thickness in these patients can result in reduced signal on near-infrared images obtained (Figure 2). An example of the effects of reduced tissue thickness is patients with wounds in areas previously treated with radiation therapy. Radiation therapy results in atrophy of the skin and soft tissue leading to better visualization of the vasculature of the underlying muscle. This results in increased signal to the area, which again can be misinterpreted as adequate perfusion for healing [19,26]. The pattern of signal obtained must be considered. If there is increased periwound signal and reduced wound bed signal, this wound is likely chronic, stalled in a dysfunctional, inflammatory state as mentioned above. The presence of slough, eschar, coagulum or placement of an advanced tissue produce in the wound bed will also result in low perfusion or tissue oxygenation saturation signals because of their effects on tissue thickness within the wound bed (Figure 3) [19]. Review of the corresponding clinical photograph obtained by the near-infrared imaging device is critical, as the image analysis provided by the device does not adjust signal reading obtained due to the presence of these tissues and products within the wound bed [27].

### 2.3. FactorsR in Variations in to Local Vasculature

#### 2.3.1. Hyperbaric Oxygen Therapy

**Figure 6:**
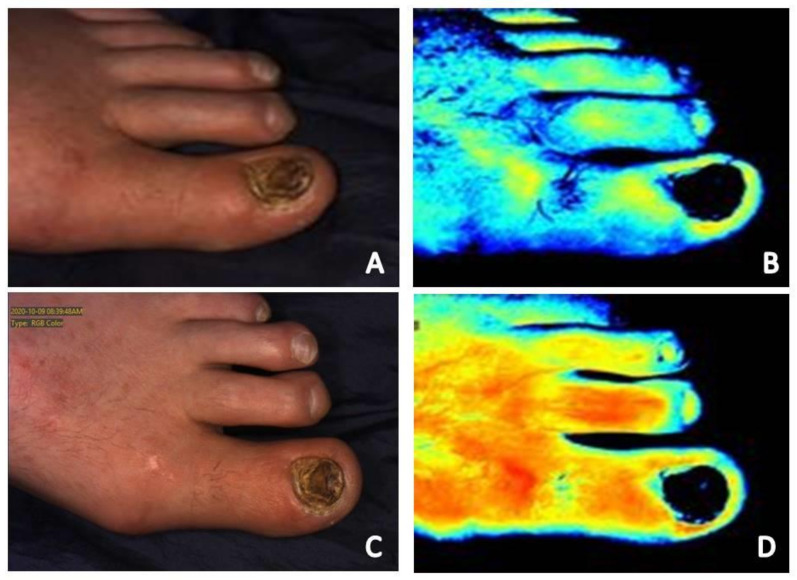
(**A**) Baseline clinical photograph; and (**B**) near-infrared image assessment of the left dorsal foot in a patient with arterial insufficiency referred by hyperbaric oxygen therapy. Note reduced signal to the dorsal foot (**C**) immediately prior to a single session of hyperbaric oxygen therapy and immediately following (**D**) this treatment.

Hyperbaric oxygen therapy works by increasing arterial oxygen pressure, resulting in vessel constriction and an oxygen gradient that prompts delivery to poorly perfused tissues [32]. The vasoconstrictive effects of hyperbaric oxygen therapy are easily visualized on comparison of near-infrared images obtains immediately prior to and following a treatment session [19]. Vasoconstriction affects mature vascular structures leading to redistribution of perfusion to area of more limited supply or newer vascular beds, i.e., areas where angiogenesis is occurring. An example of a positive response to hyperbaric oxygen therapy in a patient with arterial insufficiency is seen in Figure 6.

#### 2.3.2. Use of Local Anesthetics with Epinephrine

**Figure 7:**
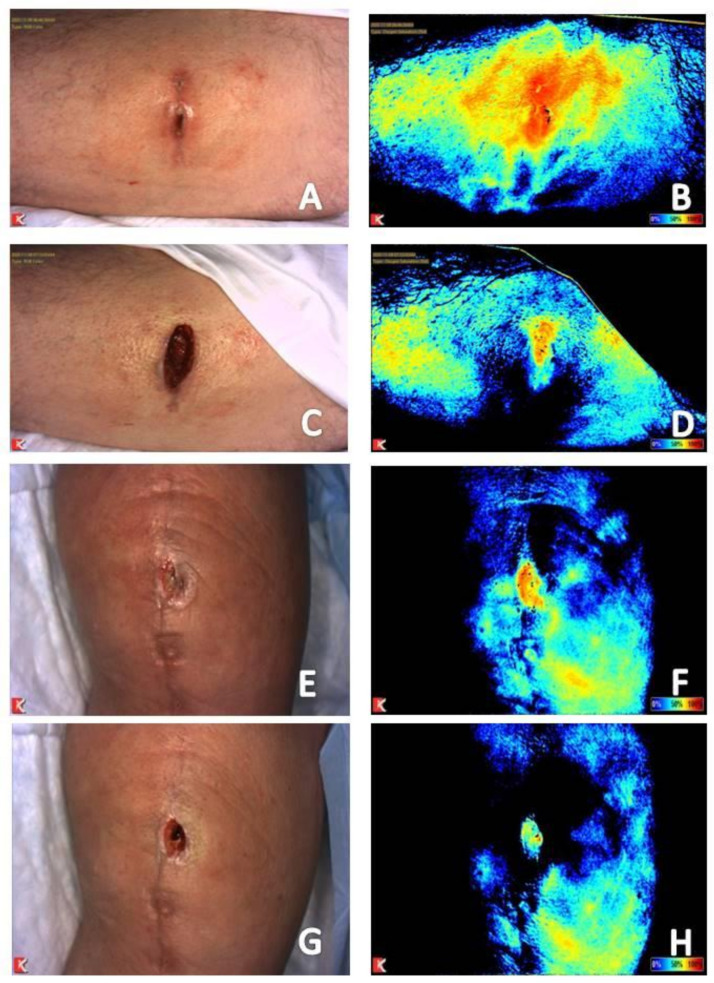
Clinical photograph and near-infrared image assessment of a wound on the abdomen and knee prior to (**A**,**B**,**E**,**F**, respectively) and following injection of local anesthetic with epinephrine (**C**,**D**,**G**,**H**, respectively) to allow for wound debridement. Note reduction in signal within the periwound area within maintenance of signal within the wound bed consistent with vasoconstriction of mature vessels in the periphery of the wound secondary to medication administered and redistribution to less mature vascular in areas where angiogenesis is occurring.

Another example of the ability to visualize vessel constriction is obtaining near-infrared images prior to and following application of local anesthetics containing epinephrine. Decreased perfusion and tissue oxygenation saturation levels will be seen at the site of injection. Figure 7 provides two examples of near-infrared images of wounds obtained prior to and following local field block with anesthetic with epinephrine. Signal reduction in the area of injection leads to vasoconstriction and shunting of perfusion to areas of more limited supply or where angiogenesis is occurring within the wound bed. The provider must bear this in mind if local anesthetics with epinephrine are used to allow for adequate debridement in patients with intact sensation to prevent resection of viable tissue. In a study of the use of fluorescence angiography to determine tissue viability in 62 breast reconstructions, a false-positive result for necrotic tissue was seen in patients where injections of local epinephrine had been performed [33].

### 2.4. Imaging Analysis Consistent with a Wound Progressing toward Resolution

#### Progression from the Inflammatory to the Proliferative Phase of Wound Healing

Both acute and chronic wounds must progress through the phases of wound healing for resolution to occur. Progression through these phases seen on near-infrared imaging differs based on whether the wound being imaged is an acute or chronic wound. An acute wound is a wound that proceeds through the four phases of wound healing—Hemostasis, inflammatory, proliferative, remodeling—In a timely fashion. A chronic wound is often delayed in a dysregulated inflammatory phase and unable to progress to the proliferative phase of healing [34]. The hallmark of the proliferative phase of wound healing is angiogenesis, production of new blood vessels necessary for nutrient delivery and waste removal [12,15]. Initial near-infrared imaging of acute wounds will show an area of reduced signal in and about the wound as the local vascular supply has been disrupted [19]. As mentioned previously, initial near-infrared imaging of chronic wounds will display increased periwound signal and reduced signal within the wound bed. Angiogenesis on near-infrared imaging will appear as an increase in signal in areas where it was previously lacking, occurring around and then within the wound bed in acute wounds [19]. In chronic wounds, periwound signal will subside while increased signal will occur within the wound bed [18,19,20]. This classic appearance of chronic wound healing on serial near-infrared imaging examination has been referred to as resembling a ray of sunlight and has been found to be a positive prognostic indicator that the wound is beginning to resolve [20]. As acute and chronic wounds resolve and enter the remodeling phase, tissue perfusion and oxygenation signals will subside back to the patient’s baseline as there is no further need for increased vascular supply to the area [19,26] (Figure 1, Figure 2 and Figure 3).

## 3. Using NIR Image Interpretation to Guide Clinical Decision-Making for Expeditious and Optimal Patient Outcomes

### 3.1. Wound Bed Preparation and Preoperative Planning

Debridement is a critical component of wound care listed in several wound-care guidelines [35]. As near-infrared imaging can discern tissue with reduced or absent vascular supply, it can be used to help guide debridement for proper wound bed preparation to expedite resolution. The use of fluorescence angiography to evaluate viable bone in patients with chronic chest wounds has been reported in two separate studies [36,37]. Fluorescence angiography helped in determination of the presence of necrotic bone, ensuring adequate debridement. Its use also helped assist in proper timing of reconstruction with a local muscle flap following removal of all necrotic tissue.

**Figure 8:**
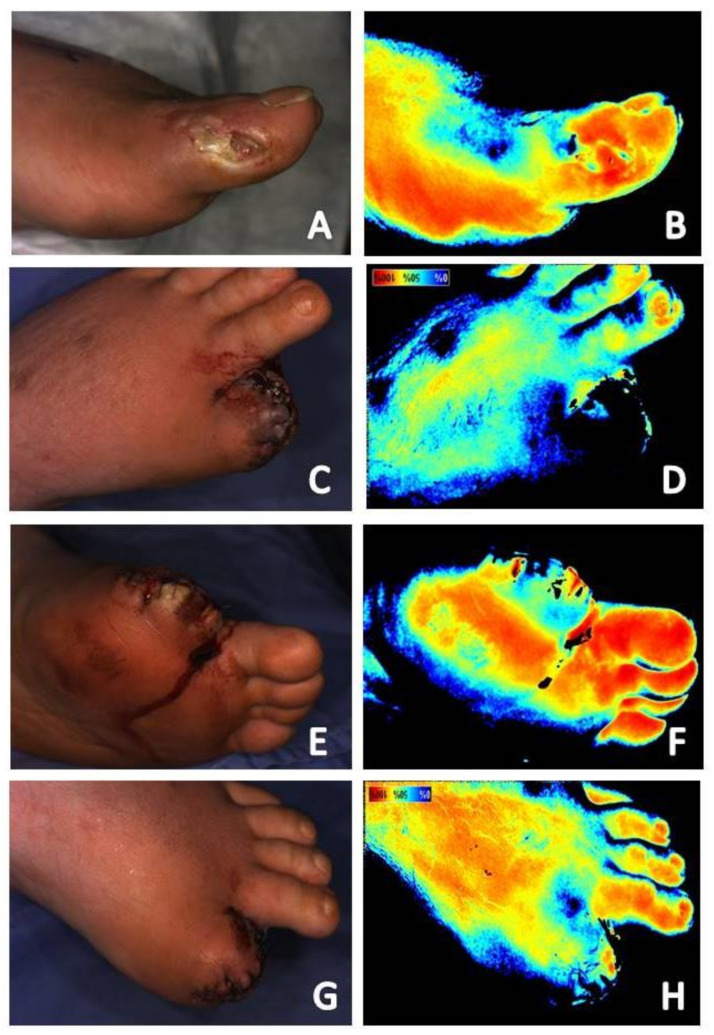
(**A**) Baseline clinical photograph; and (**B**) near-infrared image assessment of a chronic hallux wound complicated by osteomyelitis and local ischemia. Note reduced signal to the dorsal aspect of the hallux and first metatarsophalangeal joint (**B**). Clinical photograph and near-infrared image assessment of the dorsal (**C**,**D**) and plantar foot (**D**,**E**) two days following amputation, prior to the patient’s first hyperbaric oxygen therapy session. Note dorsal tissue necrosis (**C**) and reduced signal intensity (**D**) of the dorsal hallux. Necrosis of the plantar skin (**E**) was not as severe as that of the dorsal skin (**C**) following amputation. The plantar hallux had oxygenation tissue, although signal was reduced (**F**). Reduced necrosis (**G**) and increased tissue oxygenation saturation levels (**H**) were noted to the dorsal skin after ten sessions of hyperbaric oxygen therapy.

Near-infrared imaging is also able to locate perforators and provide real-time tissue assessment in selection of the appropriate perforator and corresponding flap size for use in surgical reconstruction procedures [38]. Its use in amputation flap planning may also prevent postoperative complications by ensuring selection of tissue with greater viability potential. Figure 8 shows a case of a chronic hallux wound complicated by osteomyelitis and local ischemia. Initial near-infrared imaging revealed local reduction in tissue oxygenation saturation levels on the dorsal hallux extending to the dorsal first metatarsophalangeal joint (Figure 8A,B). Amputation was performed using a fish mouth incision, which has equal tissue from the plantar and dorsal aspects of the digit. Necrosis of the dorsal flap subsequently occurred (Figure 8C,D) prompting referral for adjunctive hyperbaric oxygen therapy. The plantar flap remained with evidence of tissue oxygenation saturation (Figure 8E,F). Increase perfusion was noted to the dorsal skin with adjunctive hyperbaric oxygen therapy treatment (Figure 8G,H). If the amputation had been done using a large plantar flap, with less reliance on the dorsal skin for closure, this postoperative complication may have been avoided.

### 3.2. Supporting Information for Vascular Intervention When Routine Noninvasive Vascular Study Results Are Inconclusive or Unreliable

The ability of near-infrared imaging devices to detect local ischemia can help expedite referral to a vascular specialist for intervention. This is particularly critical in patients in which results of routine noninvasive vascular studies are consistent with adequate perfusion for healing. In a study of nine patients with chronic heel ulceration, only 2 (22.2%) had previously been diagnosed with peripheral arterial disease [27]. Routine noninvasive vascular studies in these patients were consistent with healing. However, near-infrared imaging was able to detect local ischemia to the area prompting formal vascular evaluation in eight (88.9%) of the nine patients. Vascular intervention was performed in seven (66.7%) of these patients. Those patients that had vascular intervention performed had a greater reduction in time to healing compared to those who did not (2.4 ± 0.7 vs. 29.5 ± 34.6 months). Another case report focused on the utility of near-infrared imaging to determine the presence of local ischemia in the face of improving and inconclusive results on routine noninvasive vascular studies in a chronic wound that had failed to progress toward resolution [7]. Near-infrared imaging revealed a wound with signal pattern consistent with a chronic wound stalled in the inflammatory phase of wound healing with local ischemia present. Conflicting results on routine noninvasive vascular examination prompted an expedited vascular evaluation. The patient underwent angiography revealing a popliteal aneurysm and 2-vessel runoff to the foot with a high-grade stenosis of the proximal tibial artery. Four weeks after vascular intervention, the signal pattern on near-infrared imaging reversed, consistent with transition to the proliferative phase of wound healing.

The effects of revascularization is also evident on comparison of images obtained prior to and following revascularization. Images taken prior to vascular intervention (Figure 1A,B and Figure 3C,D) showing a mottled appearance around the wound is most likely due to dilation of smaller collateral vessels attempting to provide the wound with nutrients necessary for healing. This results in shunting from other vessels and reduced flow. Following revascularization (Figure 1C,D and Figure 3E,F) the mottled appearance caused by dilation and shunting of vascular flow is improved in appearance. Tissue perfusion and oxygenation is also coalesced to the wound margin to provide nutrients to the wound bed.

### 3.3. Response to Treatment

#### 3.3.1. Resolving Infection

Serial near-infrared imaging assessment can help determine if treatment measures are effective in infection treatment. In the wound presented in Figure 8A,B, osteomyelitis of the hallux is seen as increased signal in the area of the distal phalanx of the hallux. Figure 4 shows the dorsum of the feet with increased signal present on near-infrared imaging secondary to cutaneous infection. Reduction in signal is seen as treatment is initiated and the infection resolves. Although not the focus of a previous publication, resolution of fifth metatarsal osteomyelitis was seen on near-infrared imaging following revascularization, postulated to be secondary to enhance blood flow and delivery of antibiotic therapy to the site (Figure 1) [7].

#### 3.3.2. Compression Therapy

Compression therapy is a standard of care for lower extremity ulcerations caused by venous insufficiency [31]. One confounding factor in use of this modality is patient tolerance and adherence to compression therapy use. Inadequate compression in these patients contributes to delay in healing and the high rate of recurrence of venous leg ulcerations [39]. Patient education and their understanding of the necessity of treatment have been shown to increase adherence [40,41]. Although studies on this regarding compression therapy for treatment for lower extremity ulcerations is low quality, no positive affect was found with the use of patient support groups focused on peer-support and patient-empowerment or exercise and behavior modification or additional video and written education [39]. Lack of effectiveness of these interventions may be based on the level of ability has to learn and retain the information presented. Patients with lower literacy levels, older age or cognitive compromise due to comorbidities may have difficulty in processing the information presented [42,43]. Use of personalized visual education by sharing and explaining near-infrared images prior to and after initiation of compression therapy may help improve patient understanding and subsequent adherence to use [40,41]. The positive effect of compression therapy on local tissue oxygenation saturation to a wound is seen in Figure 2. This individualized patient image along with provider education may help increase patient tolerance and adherence to treatment.

#### 3.3.3. Hyperbaric Oxygen Therapy

**Figure 9:**
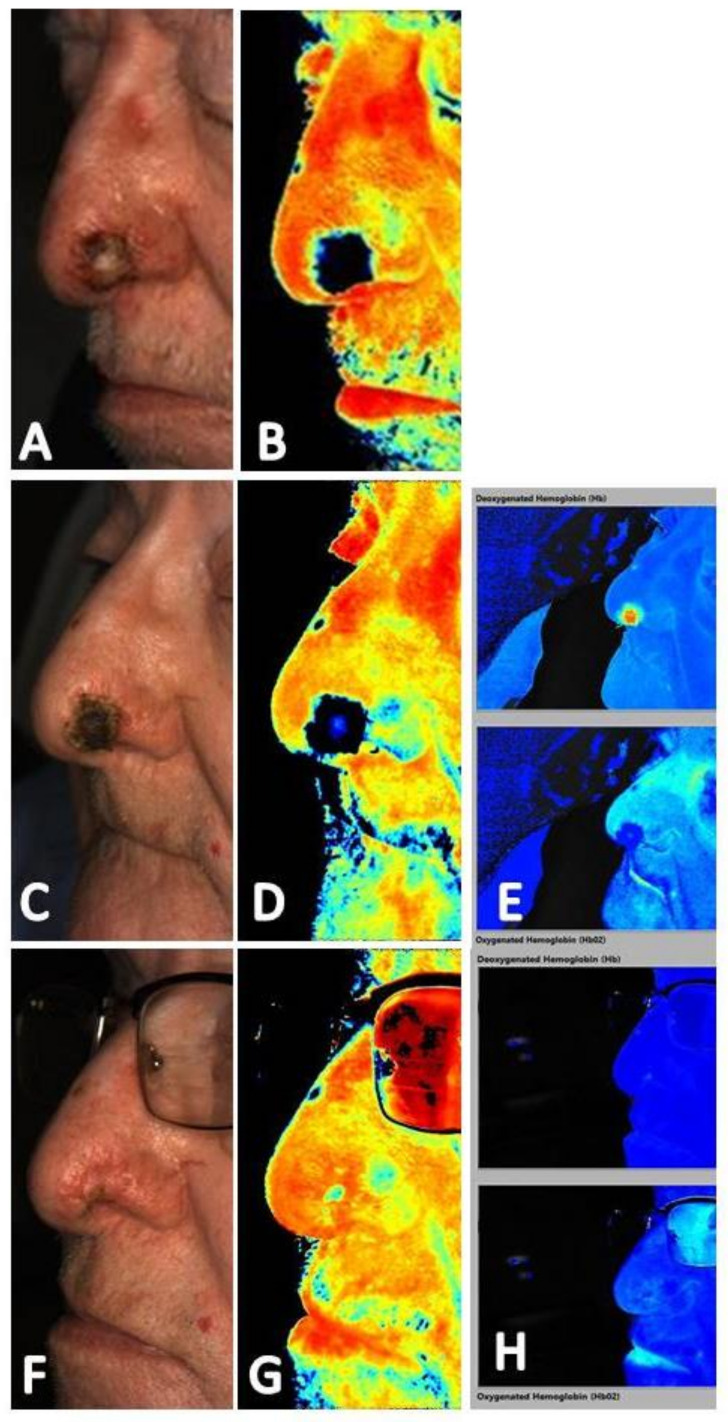
(**A**) Baseline clinical photograph; and (**B**) near-infrared image assessment of patient that had undergoing a Mohs procedure on the nose (23 July 2020). These images were taken one week prior to initiation of hyperbaric oxygen therapy. Note the absence of signal within the location of the wound on the near-infrared image (**B**). (**C**) Clinical photograph and (**D**) near-infrared image assessment following 11 sessions of hyperbaric oxygen therapy (21 August 2020). Note increase in signal seen in the center of the wound. (**E**) The corresponding deoxygenated hemoglobin (top) and oxygenated hemoglobin (bottom) views showing increased in deoxygenated hemoglobin consistent with angiogenesis occurring from the venous side within the wound. (**F**) Clinical photograph and (**G**) near-infrared image assessment following wound resolution (10 September 2020) and 19 sessions of hyperbaric oxygen therapy. (**H**) The corresponding deoxygenated hemoglobin (top) and oxygenated hemoglobin (bottom) views show an increase of oxygenated hemoglobin to the area.

**Figure 10:**
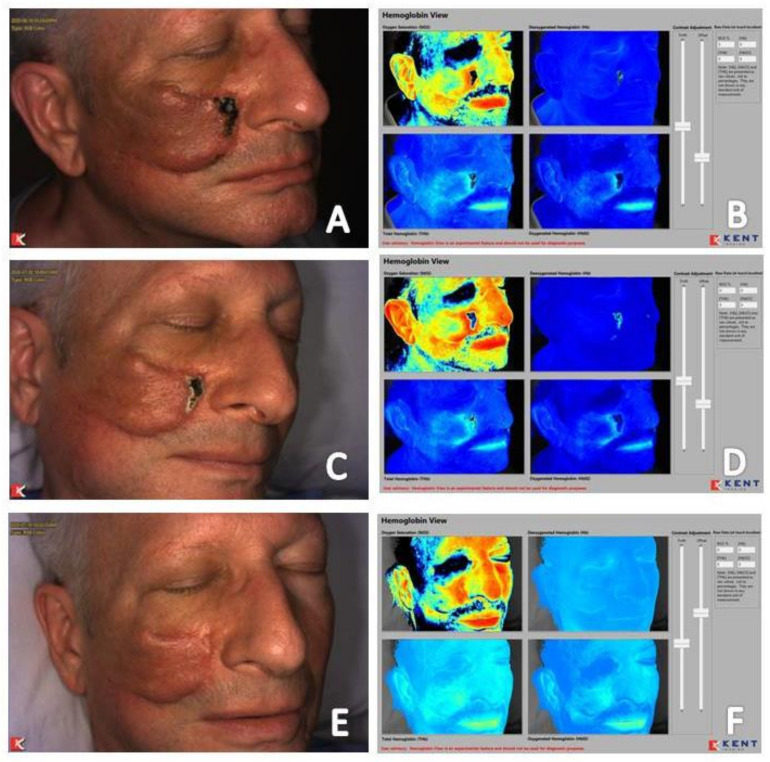
(**A**) Baseline clinical photograph; and (**B**) near-infrared image assessment, including total hemoglobin, deoxygenated hemoglobin, and oxygenated hemoglobin views, of patient that had undergone a flap for facial reconstruction following basal cell carcinoma excision (28 May 2020). Note the absence of signal within the wound bed (**B**—Oxygen saturation window) and evidence of oxygenated hemoglobin moving toward the wound from the location of the flap (**B**—Oxygenated hemoglobin window). (**C**) Clinical photograph and (**D**) near-infrared image assessment following five sessions of hyperbaric oxygen therapy. Note increase in signal within the wound bed (**D**—Oxygen saturation window) and evidence of increased deoxygenated hemoglobin within the wound bed consistent with angiogenesis occurring from the venous side within the wound (**D**—Deoxygenated hemoglobin window). (**E**) Clinical photograph and (**F**) near-infrared image assessment following wound resolution achieved after ten sessions of hyperbaric oxygen therapy. (**F**) Note increase in signal of oxygenated hemoglobin within the wound bed (**D**—Oxygen saturation window).

The utility of near-infrared imaging in patients undergoing adjunctive hyperbaric oxygen therapy for wound care has been published in several reports [19,26,37]. A study of the adjunctive use of hyperbaric oxygen therapy in the treatment of wounds of a variety of etiologies found that near-infrared imaging could identify changes in local tissue perfusion in acute and chronic wounds and enhance clinical decision-making in the selection and monitoring of patients undergoing hyperbaric oxygen therapy [19]. Maximal change in vaculogenesis and angiogenesis was noted to occur after 10 hyperbaric oxygen therapy sessions in several patients. Fluorescence angiography was used in this study, allowing for dynamic assessment of the microvasculature over the course of treatment. Imaging sequence review found that angiogenesis propagated from the venous side of the arterial system correlating with other reports that new capillaries originate from pre-existing venules within the wound bed [44,45]. This same propagation of angiogenesis from the venous side of the vascular system is also seen on non-contact, noninvasive near-infrared imaging devices. Figure 9 and Figure 10 shows patients who underwent adjunctive hyperbaric oxygen therapy following a Mohs procedure. Angiogenesis progression from the venous side of the microvasculature is clearly demonstrated as an increase in deoxygenated blood in the area of the soft tissue defect followed by an increase in oxygenated blood as resolution occurs. Imaging obtained prior to and immediately following a hyperbaric oxygen therapy session may help determine which patients will respond to this adjunctive treatment modality and tailoring of treatment to the minimum sessions required for optimal outcomes which may help improve hyperbaric oxygen therapy use and cost containment [26,37].

### 3.4. Timing of Placement and Monitoring of Response to Use of Advanced Tissue Products

Placement of advanced tissue products is an additional treatment modality that providers can use to help expedite healing. Timing of application of these products is typically based on visual inspection of the wound bed to ensure it is free of infection and non-viable tissue and has a healthy granular bed optimal for product application. Failure of response to these products leads to prolonged healing and increased healthcare cost to the facility and patient. As has been demonstrated in this document, wound beds can have a healthy appearing granular base, yet still be stalled in the inflammatory phase of healing, which may not be the optimal time for application of advanced products. The ability of near-infrared imaging to go beyond visual inspection to ensure angiogenesis of the entire wound bed through the direct, real-time visualization of tissue perfusion or tissue oxygenation saturation levels can assist in proper timing of application and efficient use [19]. Figure 2C,D shows a wound that has transition to true angiogenesis with increase tissue oxygenation saturation levels throughout the wound bed. An acellular human dermal matrix (DermACELL AWM^®^, LifeNet Health, Virginia Beach, VA, USA) was placed on the wound at that time. Continued serial assessment with near-infrared imaging allowed monitoring of progression of the wound, with continued increased tissue oxygenation saturation levels seen in the wound bed following application of the product (Figure 2E,F) which somewhat hinders inspection of the wound bed on physical examination. Use of near-infrared imaging in this way may help reduce healthcare and patient costs due to optimal timing of placement and earlier wound resolution.

## 4. Summary

Understanding images obtained with near-infrared imaging in acute and chronic wound care goes beyond the simple binary interpretation that increased signal means adequate perfusion while reduced signal means healing is unlikely to occur. This method of interpretation is a carryover from use of the technology in the operating room in which the focus is simple—Is the tissue perfused or not. In attempts to quantify results of near-infrared imaging studies and their predictive value for healing, devices and publications have attempted to correlate numeric values with results of routine noninvasive vascular studies and their predictive ability for healing [8,17,21,22,24,25]. However, this process is mired with difficulty, particularly in lower extremity vascular assessment, given that results of routine noninvasive vascular study results are not always accurate and factors beyond perfusion and oxygen can alter signals obtained with near-infrared imaging analysis.

The authors of this paper have over 10 years of combined experience with operation and image analysis of both invasive and noninvasive near-infrared imaging systems. Combined, the authors have reviewed over 20,000 images from both modalities, including images from multiple providers and facilities, in addition to using both systems to guide treatment of their own patients. This experience has resulted in identification of imaging patterns that remain consistent between both invasive and noninvasive near-infrared imaging modalities. Near-infrared images obtained provides objective information on what the phase of wound healing a wound is currently in, presence of local ischemia, presence of infection, and response to treatment. Serial near-infrared imaging assists with initial and follow-up assessment of vascular status, referral decisions, surgical planning, as well as selection and monitoring of therapeutic options including hyperbaric oxygen therapy and advanced tissue products. This all can help reduce time to healing and associated costs to the both the facility and patient as well as enhance patient quality of life.

## Data Availability

The data presented in this study are available on request from the corresponding author. The data are not publicly available due to data being part of the subjects’ medical records.
